# Constructing a disease database and using natural language processing to capture and standardize free text clinical information

**DOI:** 10.1038/s41598-023-35482-0

**Published:** 2023-05-26

**Authors:** Shaina Raza, Brian Schwartz

**Affiliations:** 1 Public Health Ontario (PHO), Toronto, ON Canada; 2 Dalla Lana School of Public Health, University of Toronto, Toronto, ON Canada

**Keywords:** Bioinformatics, Computer science

## Abstract

The ability to extract critical information about an infectious disease in a timely manner is critical for population health research. The lack of procedures for mining large amounts of health data is a major impediment. The goal of this research is to use natural language processing (NLP) to extract key information (clinical factors, social determinants of health) from free text. The proposed framework describes database construction, NLP modules for locating clinical and non-clinical (social determinants) information, and a detailed evaluation protocol for evaluating results and demonstrating the effectiveness of the proposed framework. The use of COVID-19 case reports is demonstrated for data construction and pandemic surveillance. The proposed approach outperforms benchmark methods in F1-score by about 1–3%. A thorough examination reveals the disease’s presence as well as the frequency of symptoms in patients. The findings suggest that prior knowledge gained through transfer learning can be useful when researching infectious diseases with similar presentations in order to accurately predict patient outcomes.

## Introduction

As of November 25, 2022, COVID-19 has infected more than 640 million people, with over 6.63 million deaths^1^. There are serious concerns about the impact of infectious disease on society, global health, and economy^2–4^. It is necessary to develop an efficient surveillance system that can automatically track the spread of infectious diseases by collecting, analyzing, and reporting data to those responsible for disease prevention and control.

Natural language processing (NLP) has the potential to significantly improve public health by aiding in the analysis of vast amounts of textual data from various sources^5^, including social media, electronic health records (EHRs) and published literature. By using NLP techniques, it is possible to extract valuable insights and patterns that can aid in the early detection and monitoring of infectious diseases^6^. However, challenges still exist in applying NLP to public health data, including data quality and accuracy, and variability in language and terminology used in health-related texts^7^.

To address the challenges in using free texts from EHRs and clinical notes for epidemiological and research purposes, we propose an effective NLP framework. This framework is based on deep neural network models that extract key information (entities) from the texts to study clinical and non-clinical factors associated with infectious diseases, including COVID-19. The objective of our study is to bridge the gap between NLP methods and their applications in public health to assist policymakers in decision-making and accelerate research. Our main research question (RQ) is: How can free text be transformed into a readable format to create a disease database, and query it for the factors associated with an infectious disease?

*Contributions *Our proposed framework consists of a comprehensive pipeline that includes the creation of a high-quality database from published case reports, the design and implementation of NLP models to detect and examine clinical and non-clinical concepts in the data, and a thorough evaluation process. A named entity recognition (NER) algorithm^8^ is included in the NLP models, and it is capable of accurately identifying essential clinical concepts such as diseases, conditions, symptoms, and drugs, as well as non-clinical concepts such as social determinants of health (SDOH)^9^. Furthermore, we developed a relation extraction (RE) model to identify relationships between these concepts, including disease-complication, treatment-improvement, and drug-adverse-effect associations. A two-phase evaluation approach is proposed, in which the proposed methodology is first compared to existing benchmarks, and the second phase includes a detailed analysis and human evaluation to demonstrate the framework’s usefulness for pandemic surveillance.

*Novelty of the study* The proposed NLP framework contributes to the public health domain, by introducing a data construction module, NLP modules based on Transformer^10^ architecture and a detailed evaluation phase. Through the use of few-shot learning^11,12^ techniques, our framework significantly reduces the need for manual annotations and enables more efficient and accurate identification and analysis of clinical concepts within the data. One of the major contributions of our framework is its ability to extract both the SDOHs and the clinical factors that makes it different compared to the previous works^13–19^ that have primarily focused on clinical factors. By enabling the identification of important patterns in disease diagnosis, our methodology facilitates more informed decision-making.

## Materials and methods

### Data collection

We constructed a comprehensive COVID-19 patient database using electronic case reports sourced from published literature. Specifically, we curated the case reports using a search query (Supplementary Table S1) through the National Library of Medicine (NLM)^20^ API. This study is aimed at collecting high-quality and relevant data by applying specific criteria to ensure the quality of the data collected. The participants in the study were not human subjects, but rather clinical case reports related to COVID-19 that were obtained from published literature.

The study systematically categorized case reports to analyze a diverse range of clinical experiences and interventions for COVID-19 across different demographics. The case reports were classified into five age groups: Child (6–12 years), Adolescent (13–18 years), Adult (19–44 years), Middle Aged (45–64 years), and Aged (65 + years). The collected data encompassed various approaches related to clinical classification and interventions, including screening, diagnosis, treatments, and therapies for COVID-19. The data collection period spanned from 1st March to 30th June 2022.

To ensure consistency and accuracy in the analysis, only studies published in English were included. Exclusion criteria were also applied, including excluding non-English publications, grey literature, preprints, and clinical trial registers. After applying these filtration criteria, we obtained about 5000 case reports. Each case report generally corresponds to one patient report^21^, although there may be exceptions.

### Proposed framework

**Figure 1:**
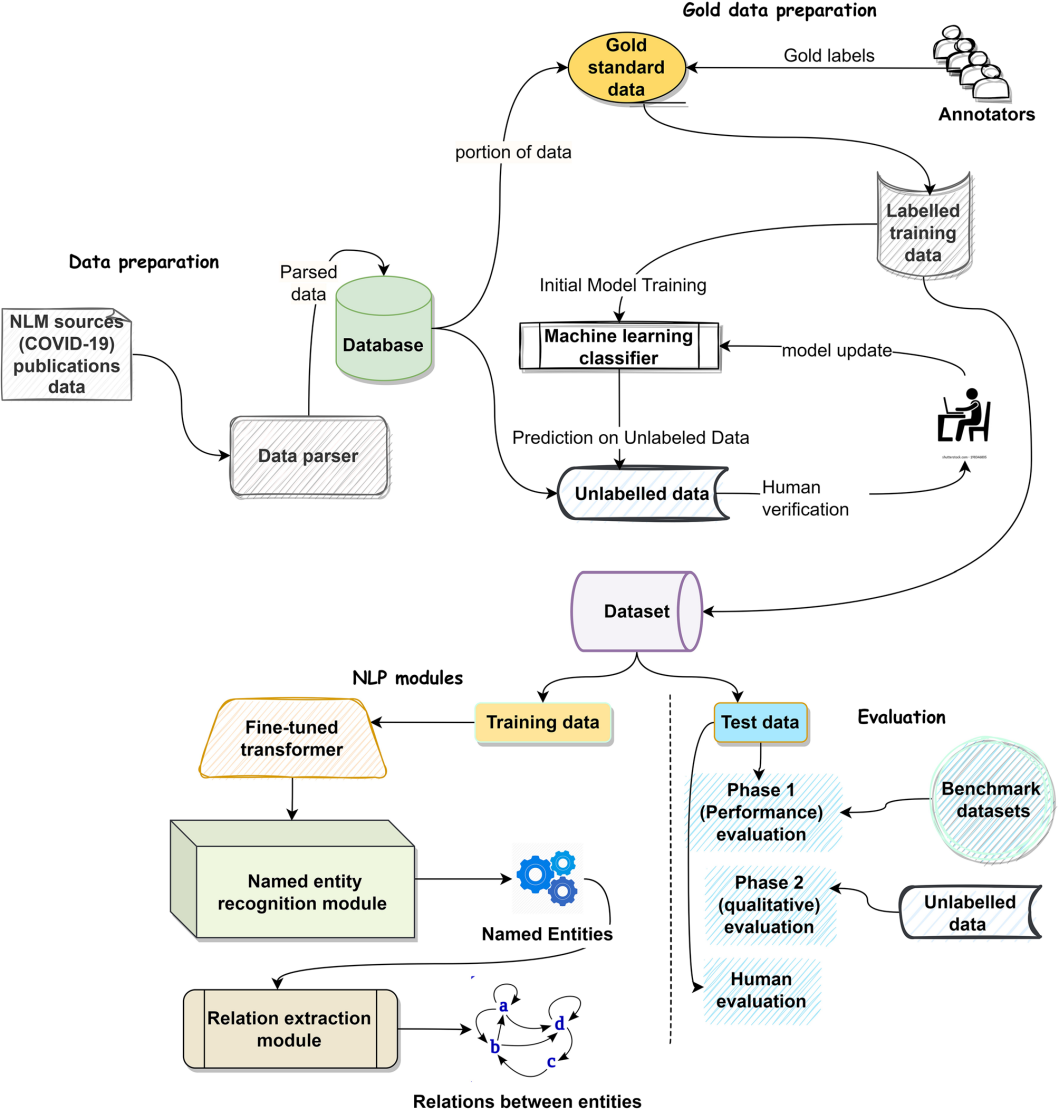
Proposed framework with data preparation, NLP modules and evaluation.

The proposed NLP framework is shown in Fig. 1 and explained next.

#### Database preparation

This study utilizes a comprehensive methodology that involved the collection of case reports from NLM sources in PDF format. These PDFs were processed using Spark OCR^22^ and transformed into a data frame format, which was then indexed with Elasticsearch^23^ to create a COVID-19 disease database.

A gold-standard dataset was created by randomly selecting 150 case reports and having four biomedical domain experts annotate them with clinical and non-clinical named entities. Approximately 550 sentences and 3,000 gold labels were produced as a result of this annotation process. A few-shot learning^11^ technique was used in conjunction with the BERT model to refine the dataset further and train deep neural network-based models. Few-shot learning refers to a machine learning approach that aims to enable models to learn from a limited amount of labeled data^11^.

The initial gold-standard dataset was used for training BERT^24^ for token classification during the few-shot learning process. Predictions were then generated for a subset of unlabeled data. New predictions were selectively sampled, a human verification is performed and then added to the existing training set. After that, the classifier was retrained on the new dataset. This iterative process was repeated until convergence was achieved. This learning loop began with 1,100 sentences from the gold-standard dataset and continued until approximately 5,000 samples were collected. This procedure yielded a maximum accuracy of around 93.5%. The final dataset included 40,000 sentences and 320,000 gold labels (named entities). Supplementary Table S2 contains key data statistics.

#### NLP models

The NLP models developed in this work are: (1) a fine-tuned Transformer model; (2) a NER module to produce named entities; (3) a RE module to define relationships between the named entities.

*Fine-tuned Transformer model* We fine-tuned the Bidirectional Encoder Representations from Transformers (BERT) for Biomedical Text Mining (BioBERT)^13^, and use our annotated dataset to prepare a fine-tuned Transformer model. Fine-tuning is a light-weight method to use the weights of an existing big language model^25^, so we prefer it over pre-training for this work.

*Named entity recognition model* The proposed NER model, shown in Supplementary Fig. S1, is an advanced adaptation of the bi-directional long short-term memory (BiLSTM)^26^ model with a conditional random field (CRF)^27^ layer added. We used a Transformer layer as the first layer to improve the model performance. This layer combines attention matrices to obtain contextualized information, which is then used to generate a word vector with varying semantics depending on the context. In this case, we make use of our task-specific Transformer model*.*

The BiLSTM layer comes after the Transformer layer, which takes the Transformer output vector as input and incorporates contextual features to derive comprehensive semantic information from the text. The output of the BiLSTM layer is the predicted label for each word in the sequence. The final layer, the CRF layer, takes the BiLSTM sequence as input and determines the dependencies between named tags. The CRF layer constrains the ultimate predicted labels using the Inside-Outside-Beginning (IOB)^28^ format, a tagging schema designed for NER chunking tasks.

The model then converts the IOB representation into a user-friendly format by associating chunks with their labels and removing NER chunks with no associated entities. The named entities are given in Supplementary Table S3, and visual representation of the named entities on piece of text is shown in Supplementary Fig. S2.

*Relation extraction model* The RE task can identify a specific relation between two co-occurring entities^29^, such as symptom-disease, disease-disease, drug-effects associations. Inspired by recent advancements in NLP related to RE^11,30–32^, we again utilize few-shot learning^11^ as a means of inferring unobserved relationships within the text. In this context, the few-shot learning enables the model to generalize and recognize novel relationships by leveraging a limited quantity of training instances from previously unseen classes^33^.

**Figure 2:**
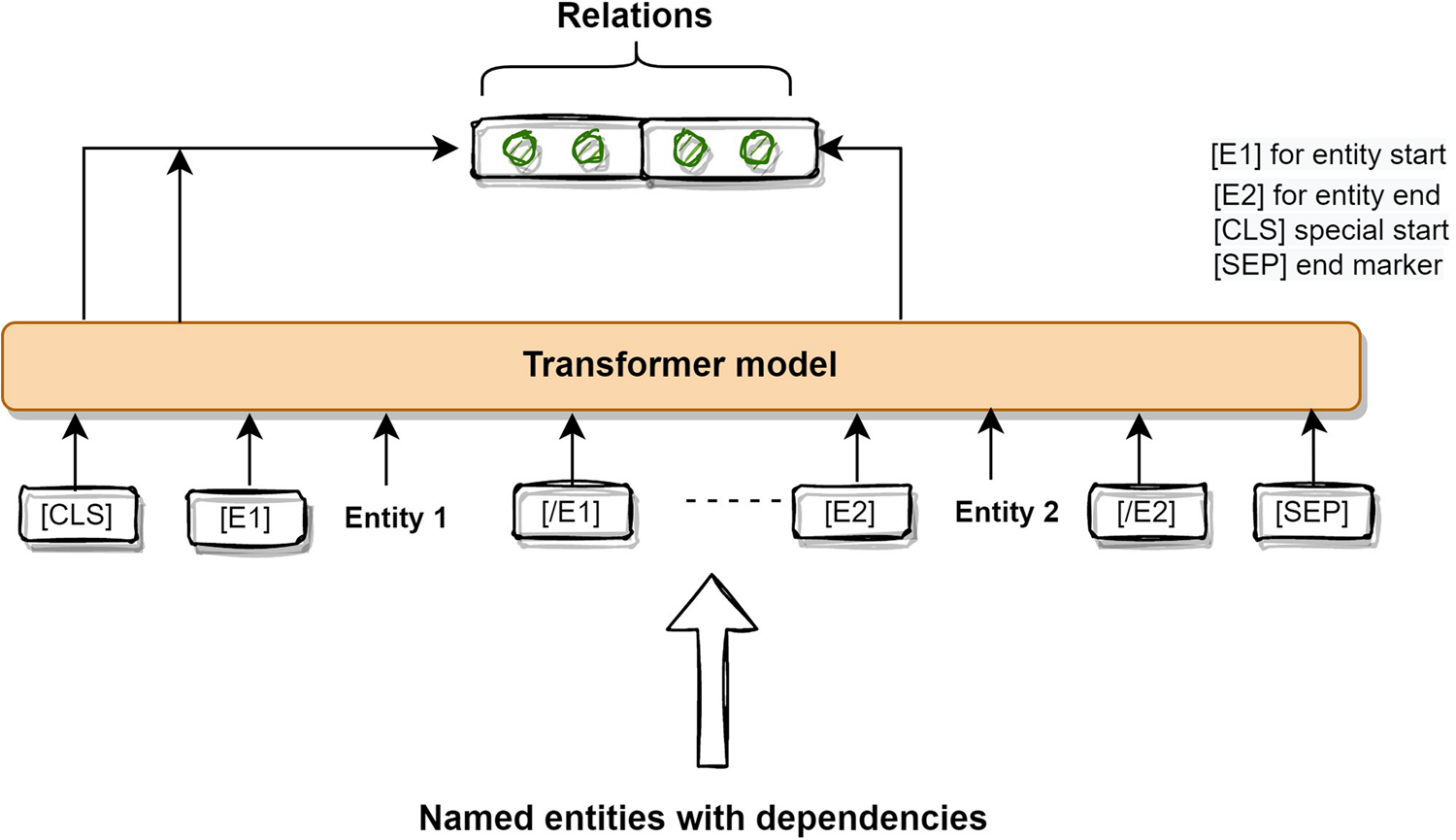
Transformer model for relation extraction.

The underlying mechanism of our proposed RE model is depicted in Fig. 2. We incorporate our fine-tuned model weights for the Transformer layer during the fine-tuning process of RE. This few-shot learning strategy embeds sentences and relationship descriptors within a unified embedding space, minimizing distances between them iteratively. As a result, the model effectively classifies unobserved relationships by leveraging the limited labelled data^34^.

#### Evaluation

Our study employs a dual evaluation strategy: Phase 1—quantitative assessment and Phase 2—qualitative assessment. We compare the accuracy of our proposed tasks with baseline approaches across benchmark datasets and demonstrate the efficiency of the proposed method for pandemic surveillance using unlabeled data. Datasets are randomly allocated into 70% training, 15% validation, and 15% testing. For our own test set, we reserve 30% of the annotated data for the evaluation purpose.

The experimental configuration utilizes an Intel(R) Core(TM) i7-8565U CPU, Google Colab Pro with cloud-based GPUs, and Google Drive for storage. Following the tradition in related works^13^, we evaluate NER and RE tasks using precision, recall, and F1-measure, reporting top results for each optimized method. BERT encoder layers are implemented using PyTorch BERT from Huggingface^35^. Human evaluation is also performed to validate the efficacy of the 2-phase evaluation strategies. Supplementary Table S4 contains benchmark dataset and baseline approach details. General hyperparameters are listed in Supplementary Table S5.

## Results

### Phase 1: quantitative assessment

In the phase 1 evaluation, the NER and RE task is compared for the performance based on F1-scores and the results are given in Table 1

**Table 1:** Results of the evaluation of the named entity recognition (NER) and relation extraction (RE) tasks using k-fold (k = 5) cross-validation on various datasets and baselines. The evaluation for NER is done on both our test set and benchmark test sets, whereas for the RE task, we do not have a labeled test set, so only benchmark test sets are used. Arrow (↑) indicates a statistically significant improvement of our proposed approach compared to other models, with a p-value < 0.05 based on a two-sample t-test.

Named entity recognition task						
Model/dataset	NCBI	BC2 GM	BC4 CHEM	i2b2-clinical	i2b2-2012	Our data
BiLSTM-CRF^36^	85.19	81.86	89.00	86.66	87.75	89.10
BILSTM-CNN-Char^26^	89.11	88.18	88.98	85.31	86.69	90.15
Att-BiLSTM-CRF^14^	85.35	84.57	91.17	89.30	86.40	89.15
MCNN^37^	85.31	81.66	88.61	87.10	84.10	89.15
CollaboNet^38^	83.09	79.96	88.61	84.80	83.70	87.39
BLUE^39^	86.62	82.47	90.62	84.62	84.75	90.46
BioBERT^13^	89.23	88.91	91.06	89.52	89.76	92.73
BioGPT^40^	90.01	88.30	89.45	88.10	88.10	89.83
Our approach	91.73↑	89.09↑	91.94↑	91.22↑	91.67↑	94.94↑

**Relation extraction task**						
**Method/dataset**	**ADE**	**Bio-Infer**	**CHEM PROT**	**i2b2- clinical**	**i2b2-2012**	**N2C2**
C4.5 DT^41^	73.30	63.67	78.66	71.11	70.04	67.35
BILSTM-CRF^42^	81.22	82.24	78.56	78.37	74.63	71.84
BiLSTM-CNN^43^	79.93	75.30	73.82	69.56	72.68	70.98
CMAN^44^	82.49	74.99	74.41	69.61	75.78	68.92
Adversarial^45^	75.17	80.59	73.84	70.78	79.73	70.60
BioBERT^13^	86.17	87.39	81.95	82.53	83.22	83.27
BioGPT^40^	91.10	88.10	83.53	81.25	82.98	84.59
Our approach	90.06 ↑	89.04	89.47 ↑	91.21 ↑	86.12	86.00

*Analysis for the NER Task:* The performance of various models for the NER task on a variety of benchmark datasets, including the test set, was assessed. As shown in Table 1, the proposed approach achieved the highest F1-scores across all datasets and significantly outperformed the baseline methods. For disease entities, a higher F1-score of 91.73% was achieved by our model on the NCBI-disease dataset. Our method, along with Bert-based methods and Att-BiLSTM-CRF, obtained F1-scores above 90% on the BC4CHEMD dataset with chemical entities. The proposed approach, BERT-based methods, and BioGPT also performed well on the named entities of proteins and genes in the BC2GM dataset. A good performance gain by our model, BERT-based and BioGPT was observed on the clinical entities provided by the i2b2 datasets. The performance gain of our approach can be attributed to the clinical embeddings provided by BioBERT that significantly improved the performance on clinical and disease entities.

The proposed NER approach achieved the highest median F1-score compared to other models in fivefold cross-validation on our test set (Supplementary Fig. S3). The F1-scores for other models ranged from 87.2 to 92.8, while our approach achieved a significantly higher median F1-score. A two-sample t-test revealed that our approach significantly outperformed most of the other baseline models for NER. Although BioBERT had higher F1-scores on some datasets, our approach still showed significant differences (p < 0.001) on our test set.

*Analysis for RE Task* As shown in Table 1, our RE method outperformed all competing methods on all benchmark datasets, demonstrating the effectiveness of the transfer learning mechanism through Transformer model. A two-sample t-test revealed that our proposed approach had a significantly higher mean F1-score of 90% when compared to all other methods tested, including BioBERT and BioGPT. To verify the statistical significance of the performance of our proposed approach on the ADE dataset, we conducted a two-sample t-test. The results showed that our proposed approach achieved a significantly higher mean F1-score of 91.73% compared to all other methods tested, including BioBERT and BioGPT (p-value < 0.05), providing additional evidence for the efficacy of our approach for the RE task. Overall, these findings indicate that our approach has real-world application potential through NLP tasks.

### Phase 2: qualitative assessment

**Figure 3:**
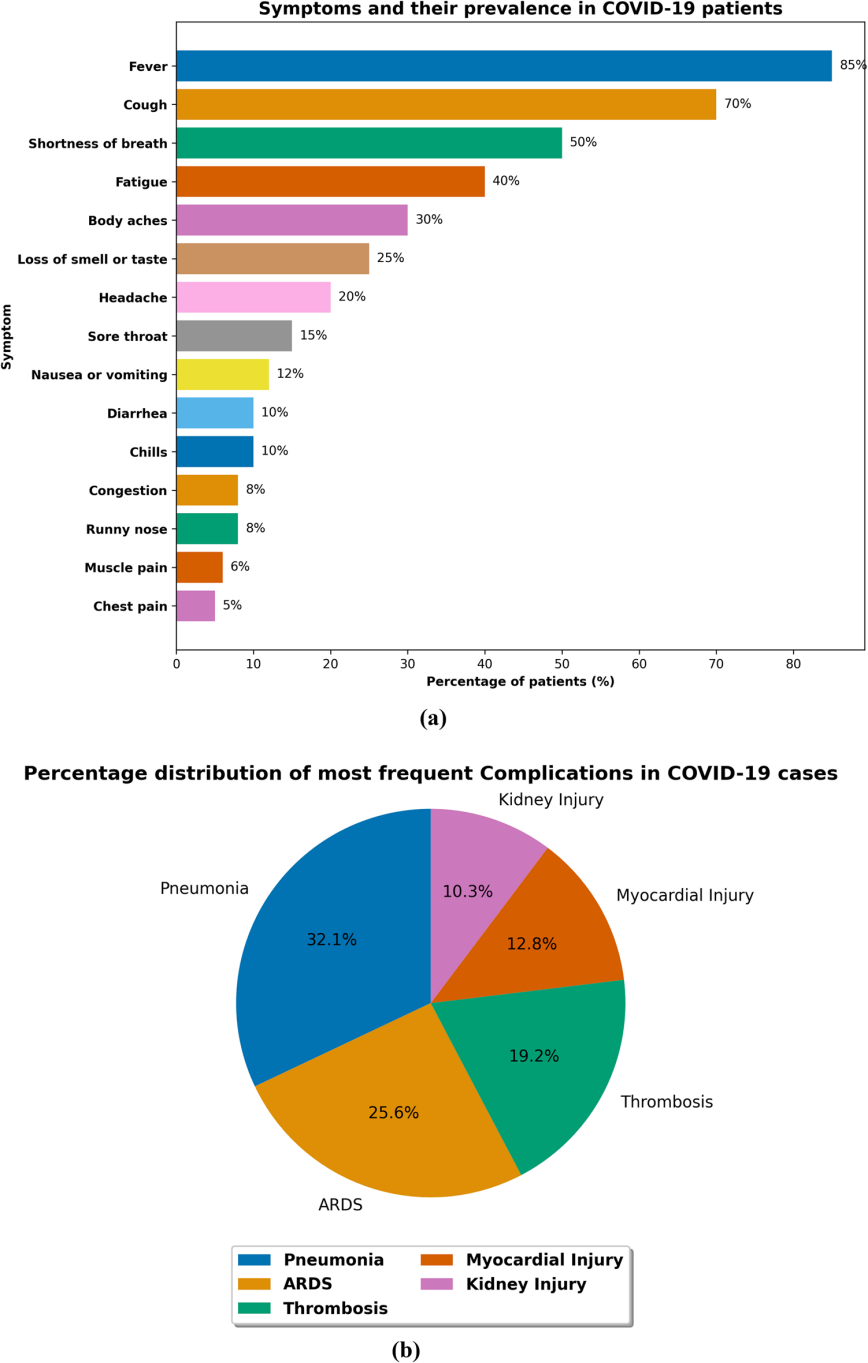
(**a**) Percentage distribution of Covid-19 symptoms in hospitalized patients, (**b**) Percentage distribution of most frequent (top-5) medical complications in hospitalized cases.

*Effectiveness of named entity recognition approach on clinical entities* We begin by showing the percentage distribution of COVID-19 symptoms among hospitalized patients in Fig. 3a and find that fever, cough, and shortness of breath are most frequent. We also show the percentage distribution of most frequent medical complications in Fig. 3b and found pneumonia, acute respiratory distress syndrome (ARDS), thrombosis, myocardial and kidney injury are among most common medical complications in COVID-19 hospitalized patients.

We further categorized symptoms by disease syndrome and present their prevalence in COVID-19 patients in Table 2. The results in Table 2 show that patients with pulmonary disease are more likely to experience cough, fever, and shortness of breath, while patients with psychological conditions are more likely to experience anxiety and depression.

**Table 2:** Prevalence of symptoms categorized according to major disease syndromes in COVID-19 hospitalized patients.

Disease Syndrome	Symptoms and Prevalence
Pulmonary	Cough (35%), Sputum production (20%), Fever (50%), Shortness of breath (45%), Fatigue (40%)
Cardiovascular	Chest pain (25%), Palpitations (20%), Shortness of breath (30%), Fatigue (15%), Dizziness (10%)
Cerebrovascular	Headache (20%), Dizziness (25%), Nausea/vomiting (15%), Altered consciousness (10%), Seizures (5%)
Psychological	Anxiety (40%), Depression (25%), Insomnia (30%), PTSD symptoms (15%), Delirium (10%)
Gastrointestinal	Abdominal pain (20%), Diarrhea (35%), Nausea/vomiting (30%), Loss of appetite (25%), Bloating (15%)

**Figure 4:**
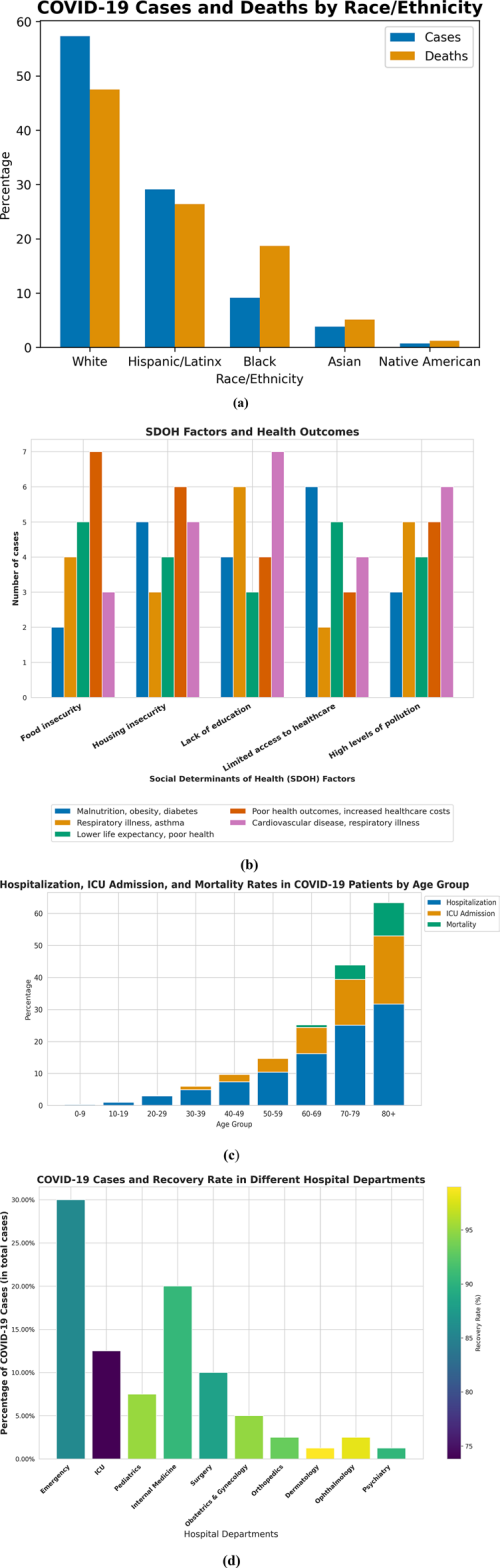
(**a**) Percentage of COVID-19 cases (y-axis) and deaths by race/ethnicity (x-axis) as reported in case reports, (**b**): Distribution of health outcomes (y-axis) for each SDOH factor (x-axis), (**c**) Hospitalization, ICU admission, and mortality rates (y-axis) in COVID-19 patients across different age groups (x-axis), (d) Relationship between COVID-19 cases (y-axis) and recovery rates in different hospital departments (x-axis).

*Effectiveness of named entity recognition approach on social determinants of health (SDOH)* The NLP framework applied to COVID-19 data also yielded SDOH-related findings depicted in Fig. 4.

Race and ethnicity were found to be significant factors associated with COVID-19 cases and deaths, with Black and indigenous communities being disproportionately affected, as shown in Fig. 4a. Socioeconomic status, health literacy, and access to healthcare were also associated with disease syndromes in COVID-19 patients, shown in Fig. 4b. Older age groups had a higher risk of hospitalization, ICU admission, and mortality, as shown in Fig. 4c, emphasizing the need for targeted interventions. The recovery rates for COVID-19 cases are shown in Fig. 4d. The recovery rate is a measure of how successful the treatment and care provided in each department have been in helping patients recover from COVID-19. These findings in Fig. 4, overall, underscore the importance of considering SDOH factors in public health surveillance and intervention efforts through NLP.

*Effectiveness of relation extraction approach* We demonstrate the effectiveness of using RE approach by specifying relationships on the run. Table 3 displays the relations of disease disorder and condition/symptom (appears afterwards). We observe in Table 3 that fever and cough, are among the most common symptoms followed by COVID-19. We also observe shortness of breath, heart failure and so, are common symptoms following hypertension.

**Table 3:** ‘Symptoms followed by disease’. Disease disorders are chosen based on the frequency of prevalence (occurring > 70%).

Disease Disorder	Following Conditions/Symptoms
COVID-19	Fever, cough, rapid breathing (tachypnea),long COVID, extensive pulmonary fibrosis.
Coronary Artery Disease	Hypertension, dyslipidemia, chest pain, myocardial infarction, heart failure
Acute Kidney Injury	Hyperkalemia, severe metabolic acidosis, hyperlactatemia, Kidney Failure, Uremia
Acute Respiratory Failure	pulmonary fibrosis, mixed venous oxygen saturation, SARS-CoV-2, Dyspnea, Hypoxia
Chest Pain	Myalgia, palpitations, shortness of breath, pressure on chest, headaches
Chronic Kidney Disease	Anosmia, ageusia, Kidney Failure, Hypertension, Anemia
Dry Cough	Rhinorrhea, nausea, vomiting, stomach Kidney Failure, Chest Infection, Asthma
Episodic Shortness of Breath	Nocturnal tachycardia, chest pain, nocturnal tachycardia, COPD, Heart Disease
Hypertension	Heart Failure, Gout, Reduced Ejection Fraction, Chronic Kidney Disease, Cardiovascular Disease
Obesity	Shortness of breath, Joint Pain, Diabetes, Hypertension, High Cholesterol
Diabetes	Peripheral Neuropathy, Increased thirst, hunger, fatigue, blurred vision, slow-healing wounds, numbness or tingling in hands or feet
Cancer	Weight Loss, Fatigue, Skin Changes, Altered Bowel/Bladder Habits, Difficulty Swallowing
Depression	Loss of interest, loss in appetite, sleep disturbance, fatigue, guilt feeling

Next, we demonstrate the relationship “DRUG causes [EFFECT]”) in Table 4. The results presented in Table 4 provide insights into the adverse effects of commonly used drugs among COVID-19 patients. For instance, persistent fever was found to be a side effect of oral amoxicillin, while trilineage hematopoiesis was associated with pirfenidone and acute headache fever was a common side effect of BNT162B2 vaccine.

**Table 4:** Relation: adverse drug events associated with common COVID-19 medications.

Drug	Effect
Oral amoxicillin	Persistent fever, sore throat, abdominal pain, loose stools, worsening rash, new-onset painful joint swelling
Pirfenidone	Trilineage hematopoiesis, swelling, erythema, ulcer, diarrhea
BNT162b2 vaccine	Acute headache, fever, nausea, vomiting, oral aphthous ulcers
Dexamethasone	Increased blood sugar, mood changes, difficulty sleeping, weight gain, muscle weakness, easy bruising
Remdesivir	Nausea, vomiting, constipation, increased liver enzymes, acute kidney injury, low blood pressure
Hydroxychloroquine	Diarrhea, nausea, vomiting, abdominal pain, retinal damage, low blood sugar
Azithromycin	Diarrhea, nausea, vomiting, abdominal pain, headache, QT prolongation, heart arrhythmias
Tocilizumab	Increased risk of infections, headache, nausea, abdominal pain, increased blood pressure, elevated liver enzymes
Ivermectin	Diarrhea, nausea, vomiting, dizziness, low blood pressure, skin rash

**Figure 5:**
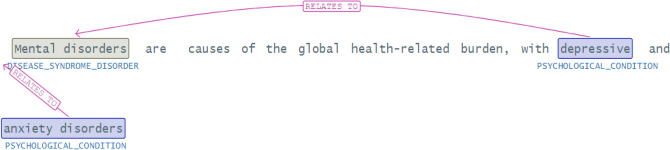
Relations in a text.

We also specify the relation between disease syndrome and psychological condition in Fig. 5 and find that depression and anxiety are the conditions in mental disorders.

Overall, these results show that our proposed NLP framework also has the potential for RE, which can aid in identifying and tracking the spread of infectious diseases and their associated risk factors.

### Human evaluation

To further assess the performance of our proposed approach for the NER and RE tasks, we conducted a human evaluation. We chose 100 documents at random from the NCBI-Disease dataset and 50 documents from our test set for the NER task. Three domain experts annotated the documents, and the inter-annotator agreement^46^ was calculated using Fleiss’ kappa^47^, which revealed significant agreement (kappa score of 0.75). Our proposed method outperformed all other baseline methods, with an average precision of 89%, recall of 91%, and F1-score of 90%.

Next, we chose 50 documents at random from the ADE dataset and 50 documents from the BioInfer dataset for the RE task. Three domain experts annotated the documents, and the inter-annotator agreement was calculated using Fleiss’ kappa, which revealed significant agreement (kappa score of 0.73). Our proposed method outperformed all other baseline methods, with an average precision of 86%, recall of 88%, and F1-score of 87%.

These findings indicate that our proposed method is highly effective for both NER and RE tasks, as evidenced by quantitative and qualitative evaluations.

## Discussion

*Principal findings* In this study, we successfully constructed a dataset and inferred valuable information to address our research question. Our approach enables the creation of a dataset from unstructured text, preparing it to study infectious diseases such as COVID-19. Although we focus on COVID-19 data, the methodology can be applied to various diseases. The disease database we developed serves as a critical resource for pandemic surveillance, with common COVID-19 symptoms such as pneumonia, respiratory infections, ARDS. Furthermore, we identified relationships between drugs and diseases. This framework benefits clinicians, medical professionals, nurses, epidemiologists, and researchers by streamlining data acquisition and decision-making.

Our experiments highlighted the impact of transfer learning in detecting COVID-19-related entities and relations. Both our NER and RE methods with pre-trained embeddings from Transformer architectures showed improvements over baseline methods. Additionally, few-shot learning proved useful in reducing annotation costs for building models, though further exploration of techniques for large-scale re-annotation is recommended. We also attempted to predict unseen relationships in texts using NLP. However, this approach differs from extracting causal relationships typically used in epidemiological studies. We suggest incorporating the Bradford Hill^48^ criteria and aligning public health initiatives with RE tasks. We also suggest using BioGPT^40^ or GPT-2^49^ for RE as well as NER experiments to see if it makes an improvement.

*Error analysis* Our error analysis revealed that our model struggled to recognise certain abbreviations in the NER task. In particular, in the BC2GM dataset, our model had low recall for abbreviations such as "RNA" and "DNA." This is most likely because these abbreviations have multiple meanings and can be used in a variety of contexts. Furthermore, we discovered that our model struggled to distinguish between similar entities in the NCBI-Disease dataset. For example, the model frequently mixed up the terms "glioma" and "lymphoma," which both refer to cancer types. This implies that our model could benefit from more training data that highlights the subtle differences between these types of entities.

*Limitations* Limitations of our study include the reliance on published case reports, which may result in a biased sample towards sicker, hospitalized patients with Long-COVID, and those seen by academic physicians. This excludes milder cases and patients who may be underserved or live in remote areas. Furthermore, our NLP approach to extracting relationships among entities may identify coincidental associations rather than causal links. Further research on causality criteria in public health is necessary. Despite the limitations of the study, the paper provides useful insights for clinicians, medical professionals, nurses, epidemiologists, and researchers, while further research on causality criteria in public health is necessary.

## Conclusions

This study demonstrates that NLP-based methods can be used to identify the presence of disease, symptoms, and risk characteristics from the free-text data. Transfer learning is promising for developing predictive disease models with limited data. The proposed methodology provides a robust way to infer named entities and relations in the texts. Over the state-of-the-art methods, the proposed methods achieve better performance on F1-score for tasks. The current study also shows the effectiveness of the proposed approach for pandemic surveillance. Further studies are needed to validate the effectiveness of our approach in different clinical contexts and with larger and more diverse datasets. In addition, it would be interesting to explore the potential of our method in other applications, such as in real-time monitoring of disease outbreaks or tracking the progression of pandemics across different geographic locations.

## Supplementary Information

Supplementary Information.

## Data Availability

The data underlying this article will be shared on reasonable request to the corresponding author.
