# Supplementary material for: Constructing a disease database and using natural language processing to capture and standardize free text clinical information
Source: Sci Rep. 2023 May 26;13:8591. doi: 10.1038/s41598-023-35482-0 (PMC10215040; doi:10.1038/s41598-023-35482-0)

**Constructing a disease database and using natural language processing to capture and standardize free text clinical information**

Shaina Raza ^1,2*,^ PhD; Brian Schwartz ^1,2^, MD, MScCH

^1^ Public Health Ontario (PHO),

Toronto, ON, Canada.

^2^ Dalla Lana School of Public Health, University of Toronto,

Toronto, ON, Canada.

Corresponding Author: [shaina.raza@utoronto.ca](mailto:shaina.raza@utoronto.ca)

**Supplementary file**

**Supplementary Table S1:** Search query for data cohort

| Search: COVID AND LONG COVID  Filters: Free full text, Case Reports, English, Child: 6-12 years, Adolescent: 13-18 years, Adult: 19-44 years, Middle Aged: 45-64 years, Aged: 65+ years.  COVID: "sars-cov-2" OR "covid" OR "covid-19"  LONG COVID: "post-acute COVID-19 syndrome" OR "long covid"  Query: ((("sars-cov-2" OR "covid" OR "covid-19") AND ("post-acute COVID-19 syndrome" OR "long covid")) AND ("case reports") AND ("english") AND ("child" OR "adolescent" OR "adult" OR "middle aged" OR "aged")) |
| --- |

**Supplementary Table S2.** Natural language processing-based summary of COVID-19 cohort.

| The study encompassed a total of 5,000 patients, divided into five age groups: 6-12 years, 13-18 years, 19-44 years, 45-64 years, and 65+ years. The patient population consisted of 45% males (n=2,250), 31% females (n=1,550), and 24% with unidentified gender (n=1,200). These patients were admitted through various channels, such as operating rooms, emergency rooms, ICUs, and other hospital settings.  The patients were diagnosed with several disease syndromes, including cardiovascular, cerebrovascular, respiratory, long-COVID, neurological, multisystem disease, acute respiratory distress syndrome (ARDS), myalgic encephalomyelitis/chronic fatigue syndrome (ME/CFS), trauma, and other diseases. Co-existing disorders with COVID-19 comprised cardiovascular disease and hypertension, chronic respiratory disease and pulmonary hypertension, diabetes-obesity, diabetes-hypertension, diabetes-hyperlipidemia, hypertension-obesity, coronary artery disease (CAD)-diabetes, cerebrovascular and hypertension, pneumonia and acute respiratory distress syndrome (ARDS), and other comorbidities.  Clinical outcomes covered confirmed positive cases, diagnoses, hospitalizations, mortalities, recoveries, and cases with unidentified outcomes (as demonstrated through the figures in the analysis. |
| --- |

**Supplementary Figure S1**: Proposed Named entity recognition model.


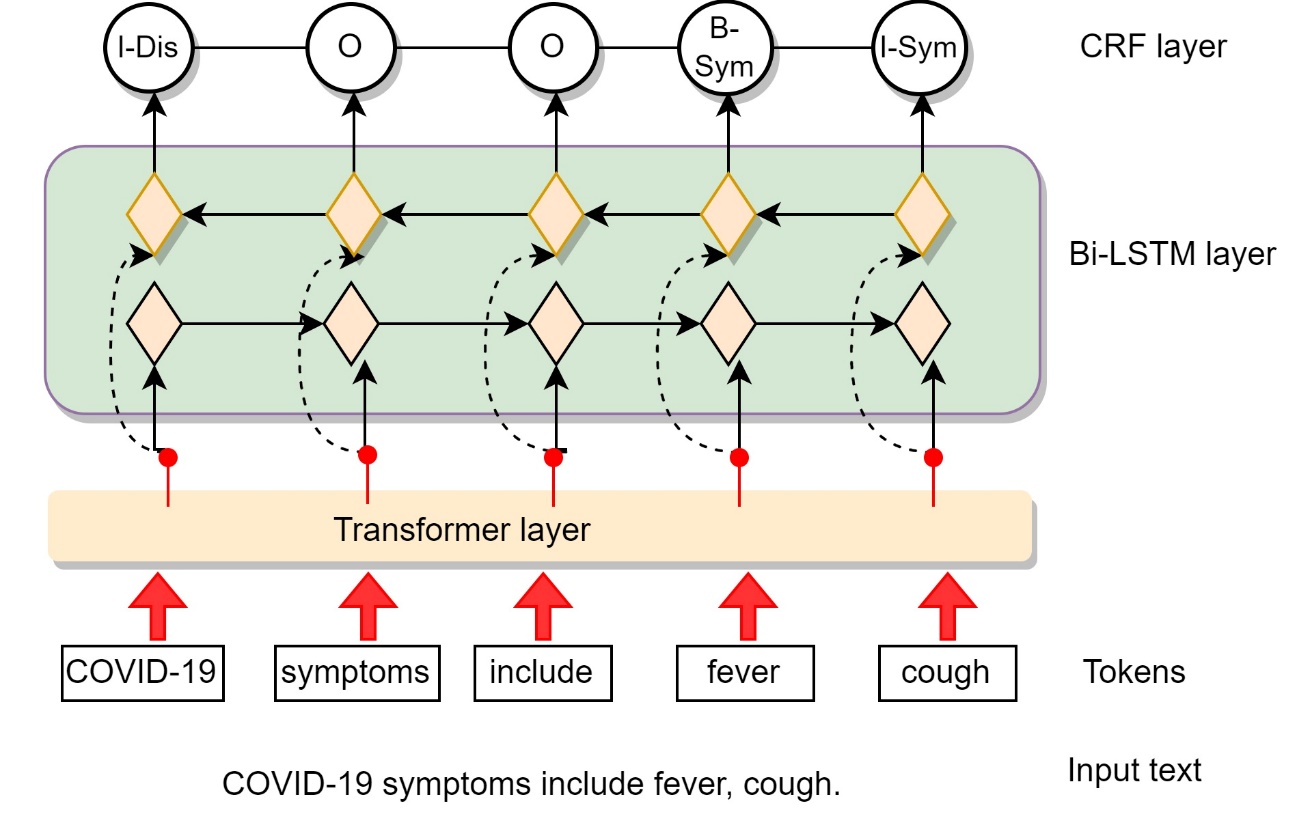


**Supplementary Table S3:** Named entities, the inspiration of these named entities is taken from JohnSnowLabs [1].

| GENDER  HEIGHT  AGE  DATE  WEIGHT  SMOKING  RACE_ETHNICITY  RELATIVE_DATE  DRUG_NAME  DURATION  ADMISSION_DISCHARGE  ALCOHOL  SUBSTANCE  EMPLOYMENT  TIME | OXYGEN_THERAPY  HEART_DISEASE  CLINICAL_DEPARTMENT  BLOOD_PRESSURE  DISEASE_SYNDROME  DOSAGE  TREATMENT  TEST  PSYCHOLOGICAL_CONDITION  SYMPTOM  RESPIRATION  LABOUR_DELIVERY  INTERNAL_ORGAN  EXTERNAL_BODY_PART  PROCEDURE | DIABETES  VACCINE  HYPERLIPIDEMIA  HYPERTENSION  DEATH_ENTITY  SYMPTOM  RESPIRATION  LABOUR_DELIVERY  TEMPERATURE  KIDNEY_DISEASE  OBESITY  BMI  PULSE  INJURY_OR_POISONING |
| --- | --- | --- |

**Supplementary Figure S2:** Case study, Visual representation of named entities from the snippet of case report.


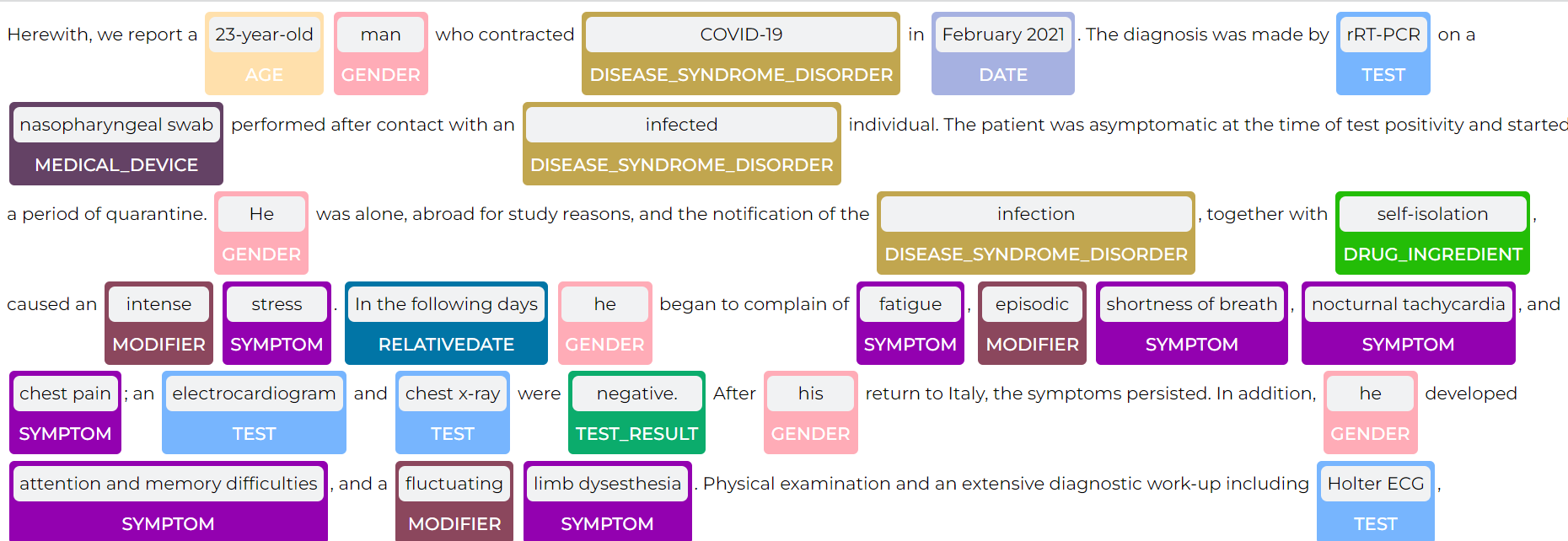


**Supplementary Table S4:** Benchmark datasets and methods.

| **Corpus** | **Entity Types** | **Data Size** |
| --- | --- | --- |
| **NER Task** | | |
| NCBI-Disease | Diseases | 793 PubMed abstracts |
| BC2GM | Gene/Proteins | 20,000 sentences |
| BC4CHEMD | Chemicals | 10,000 PubMed abstracts |
| i2b2-Clinical | Problem, Treatment, and Test. | 426 discharge summaries |
| I2b2 2012 | Clinical (problems, tests, treatments, clinical departments, occurrences, and evidence) | 310 discharge summaries |
| **RE Task** | | |
| ADE | Drugs; Adverse Effects; Dosages | 2,972 MEDLINE case reports |
| BioInfer | Protein-Protein Interaction | 1,098 sentences |
| CHEMPROT | Protein-Chemical | 1,820 PubMed abstracts |
| i2b2-Clinical | TrIP, TrWP, TrCP, TrAP, TrAP, TeRP, TeCP, PIP | 426 discharge summaries |
| i2b2 2012 | Before, After, Simultaneous, Begun by, Ended by, During, Before Overlap (temporal relations) | 310 discharge summaries |
| N2C2 | Strength-Drug (Severity), Form-Drug (Form), Dosage-Drug (Do), Frequency-Drug (Fr), Route-Drug (Route), Duration-Drug (Du), Reason-Drug (Reason), ADR-Drug (Adverse) | 288 longitudinal patient records |
| **Baseline methods for NER task** | | |
| BiLSTM-CRF | Bidirectional LSTMs and CRF architecture | NER |
| BiLSTM-CNN-Char | Hybrid (LSTM) and Convolutional Neural Network (CNN) architecture | NER |
| Att-BiLSTM-CRF | Attention-based BiLSTM model with a CRF layer | Chemical NER |
| MCNN | Multiple (M) label CNN-based network | Disease NER from biomedical literature |
| CollaboNet | Collaboration of deep neural networks (BiLSTM-CRF) with a single task model trained for each specific entity type | NER |
| BLUE | Biomedical Language Understanding Evaluation (BLUE) with BERT-based pre-training | Biomedical language representation tasks |
| BioBERT | Pre-trained biomedical language representation model | Biomedical text mining |
| BioGPT | Variant of the Generative Pre-trained Transformer (GPT) language model specifically trained on biomedical and clinical text | RE |
| **Baseline methods for RE task** | | |
| C4.5 DT | Statistical technique using decision tree method | Causal relations |
| BiLSTM-CRF | BiLSTM architecture | RE from biomedical literature |
| BiLSTM-CNN | Hybrid BiLSTM+CNN model with pretrained word embedding and shortest dependency path embedding | Protein-protein extraction |
| CMAN | Deep cross-modal attention network | Joint entity and relation extraction |
| Adversarial (Adv) training | Deep neural network for joint extraction of entities and relations |  |
| BioBERT | Pre-trained biomedical language representation model | RE |
| BioGPT | Variant of the Generative Pre-trained Transformer (GPT) language model | RE |

**Supplementary Table S5:** Hyperparameter and best result value (values in parenthesis represent the parameter ranges tested)

| **Parameters for NER** | |
| --- | --- |
| **Hyperparameter** | **value** |
| LSTM state size | 200 [200 - 300] |
| dropout rate | 0.5 [0.2 - 0.7] |
| Epochs | 40 [20- 80] |
| Batch size | 16 [8 - 128] |
| Learning rate (lr) | 1.e-05 [1.e-9 – 1.e-2] |
| lr decay coefficient (po) | 0.005 [0.001, 0.01] |
| Warmup steps | 10,000 |
| Optimizer | ADAM, β1=0.9 and β2=0.999 |
| Word dimension | 300 [50 – 450] |
| Hidden size LSTM | 300 |
| Gradient clipping | 5.0 |
| **General parameters for Transformer-based models** | |
| The general parameters used for fine-tuning transformer-based architectures (BioBERT and others) are maximum sequence length of 128, number of layers as 12, number of attention heads also 12 and embedding size as 768. For different datasets, the fine-tuning takes different hours (2 hours, 3 hours, 4 hours and 10 hours for our dataset). In the NER task, we fixed the length of sentences to 512, whereas, for the RE task, we use a sentence length of 128 in our experiments. | |

**Supplementary Figure S3**

**Figure S3 (a):** Comparison of F1-scores for various models (x-axis) on 5-fold Cross-validation for the NER task.


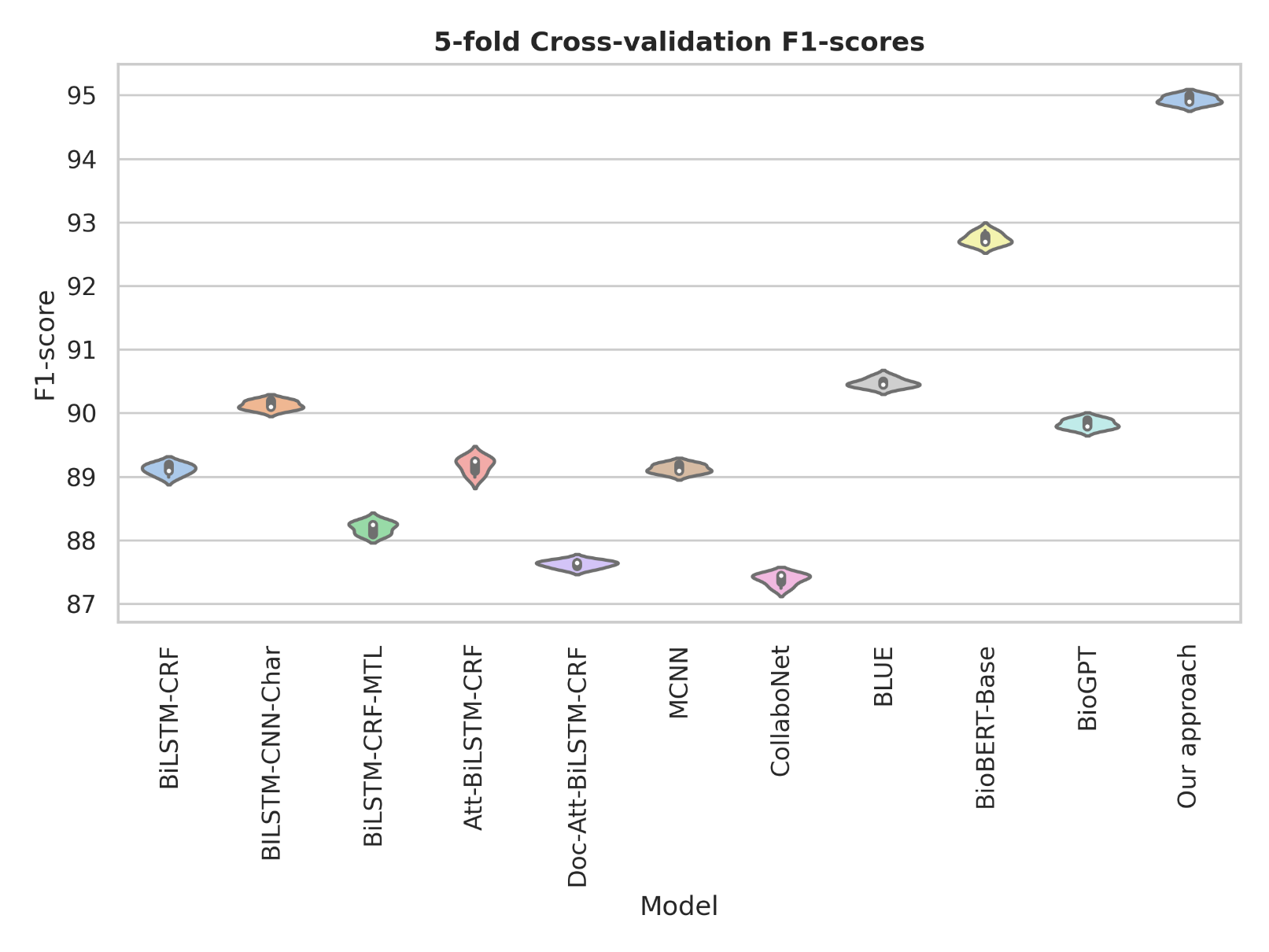


**Figure S3 (b):** Comparison of F1-scores for various models (x-axis) on 5-fold Cross-validation for the RE task.


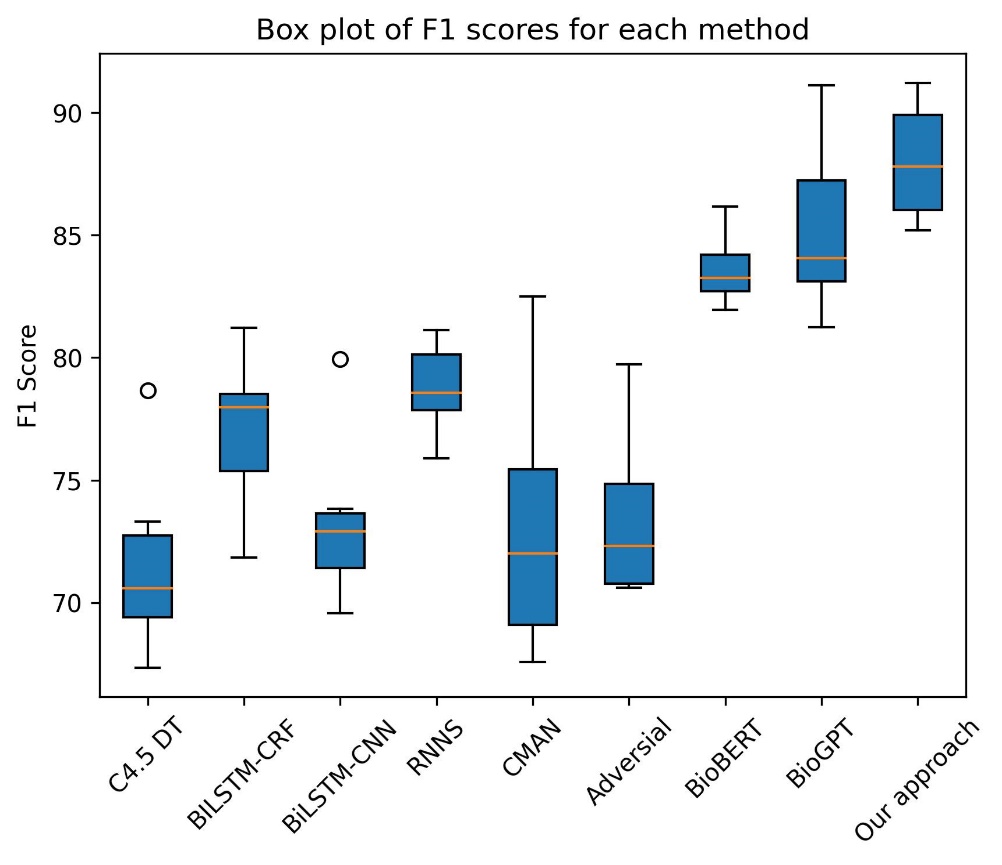

Supplement: Supplementary file 1 — Supplementary Information. [file 41598_2023_35482_MOESM1_ESM.docx]
